# Early differential diagnosis model for acute radiation pneumonitis based on multiple parameters

**DOI:** 10.1042/BSR20200299

**Published:** 2020-04-17

**Authors:** Zhiwu Wang, Qiong Wu, Liang Dong, Haoyu Fu, Qiwei Liu

**Affiliations:** 1 Department of Chemoradiotherapy, Tangshan People’s Hospital, Tangshan, P.R. China; 2 Nuclear Medicine Laboratory, Tangshan People’s Hospital, Tangshan, P.R. China

**Keywords:** Diagnosis model, differential diagnosis, infectious pneumonia, radiation pneumonitis, radiotherapy

## Abstract

**Objective:** The present study aimed to construct a diagnosis model for the early differentiation of acute radiation pneumonitis (ARP) and infectious pneumonitis based on multiple parameters.

**Methods:** The present study included data of 152 patients admitted to the Department of Radiochemotherapy, Tangshan People’s Hospital, who developed ARP (91 patients) or infectious pneumonia (IP; 61 patients) after radiotherapy. The radiophysical parameters, imaging characteristics, serological indicators, and other data were collected as independent variables, and ARP was considered as a dependent variable. Logistics univariate analysis and Spearman correlation analysis were used for selecting independent variables. Logistics multivariate analysis was used to fit the variables into the regression model to predict ARP.

**Results:** The univariate analysis showed that the positional relation between lesions and V20 area (PRLV), procalcitonin (PCT), C-reactive protein (CRP), mean lung dose (MLD), and lung volume receiving ≥20 Gy (V20) correlated with ARP while the planning target volume (PTV) dose marginally correlated with ARP. The multivariate analysis showed that the PRLV, PCT, white blood cell (WBC), and MLD were independent diagnostic factors. The nomogram was drawn on the basis of the logistics regression model. The area under the curve (AUC) of the model was 0.849, which was significantly better than that of a single indicator and the sensitivity and specificity of the model were high (82.4 and 82.0%, respectively). These results predicted by the model were highly consistent with the actual diagnostic results. The decision curve analysis (DCA) demonstrated a satisfactory positive net benefit of the model.

**Conclusion:** The diagnosis model constructed in the present study is of certain value for the differential diagnosis of ARP and IP.

## Introduction

Acute radiation pneumonitis (ARP) is the main dose-limiting toxic reaction after chest radiotherapy, with a prevalence of up to 23.9% [1]. It greatly impacts the normal treatment, quality of life, and survival of patients [2]. Early treatment can effectively prevent the progression of the disease and avoid adverse consequences, but it depends on the accurate diagnosis of APR in the early stage. The diagnosis of ARP requires a history of radiotherapy, changes in imaging, relevant symptoms, exclusion of other types of pneumonitis, and so on [3]. However, the diagnostic criteria had the following drawbacks: (1) they were not suitable for the early diagnosis of ARP. Due to the low specificity of imaging and symptoms of ARP, it was necessary to exclude many lung diseases with similar manifestations, mainly including bacterial, fungal, and other infectious pneumonia (IP) [4]. The optimal method was to obtain an accurate pathogenic diagnosis, which nonetheless normally took a long time and tended to delay the timing of early diagnosis and treatment. Therefore, the introduction of relevant markers in diagnostic criteria that could quickly and accurately exclude IPs was critical for the early and accurate diagnosis. Previous studies demonstrated that procalcitonin (PCT) could effectively distinguish bacterial IP from ARP, and thus recommended to include PCT in the criteria as a diagnostic factor. (2) At present, a large number of studies have confirmed the existence of a variety of risk factors for ARP [5,6]. Nonetheless, these factors are still not listed in the current diagnostic criteria. Hence, including these relevant risk factors may further improve the accuracy of the diagnosis. (3) The criteria only contained a list of items, which are described in general and lack specific details. Furthermore, a score-based objective diagnosis system has not been established yet. Therefore, constructing a diagnosis model by integrating multidimensional information using multivariate analysis may be more beneficial for clinical applications. The present study aimed to retrospectively analyze data of patients who developed pneumonitis after radiotherapy, so as to initially construct a model for the early diagnosis of ARP using the multidimensional diagnostic information, and achieve visualization.

## Materials and methods

### Patients and general information

Data of patients hospitalized due to pneumonitis in the Department of Radiochemotherapy, Tangshan People’s Hospital, between 1 January 2014 and 30 June 2017, were retrospectively analyzed. The inclusion criteria were as follows: (1) patients were pathologically diagnosed with lung cancer. (2) Patients underwent chest radiotherapy within 6 months. (3) Patients developed symptoms of acute pneumonitis, such as fever, cough, and dyspnea, and were admitted to hospital within 3 days of onset. (4) Chest computed tomography (CT) suggested intrapulmonary inflammatory lesions. (5) Patients underwent PCT examination immediately after admission. The exclusion criteria were as follows: (1) symptoms were caused by tumor progression, aggravated chronic obstructive pulmonary disease (COPD), heart diseases, pulmonary embolism, anemia, and so on. (2) All patients had been exposed to patients with pneumonia or influenza-like illness during the preceding 7 days or had resided in an area where influenza virus was being spread.

Based on the inclusion and exclusion criteria, a total of 159 patients were enrolled in the initial screening, from which 7 patients were excluded due to complicated conditions and unclear diagnosis. Ultimately, 152 patients were selected, including 112 men and 40 women, aged 36–82 years (median age: 62 years) (Table 1).

**Table 1:** Univariate analysis of clinical characteristics of categorical variables

Characteristics	Number of patients	ARP n(%)	IP, *n* (%)	*OR* (95% CI)	*P*-value
Total	152	91(59.9)	61(40.1)		
Age (years)					
<65	78	50 (64.1)	28 (35.9)	1	
≥65	74	41 (55.4)	33 (44.6)	0.696 (0.363–1.334)	0.274
Sex					
Female	112	64 (57.1)	48 (42.9)	1	
Male	40	27 (67.5)	13 (32.5)	1.558 (0.728–3.331)	0.253
Pathological diagnosis					
Adenocarcinoma	53	30 (56.6)	23 (43.4)	1	
Squamous carcinoma	39	25 (64.1)	14 (35.9)	1.369 (0.585–3.204)	0.469
Small cell carcinoma	50	28 (56.0)	22 (44.0)	0.976 (0.448–2.127)	0.951
Other types	10	8 (80.0)	2 (20.0)	3.067 (0.594–15.840)	0.181
Stage					
II–III	103	66 (64.1)	37 (35.9)	1	
IV	49	25 (51.0)	24 (49.0)	0.584 (0.293–1.164)	0.126
Ps score					
≤1	94	55 (58.5)	39 (41.5)	1	
2–3	58	36 (62.1)	22 (37.9)	1.160 (0.593–2.269)	0.664
PRLV					
In V20 area	109	82 (75.2)	27 (24.8)	1	
Out of V20 area	43	9 (20.9)	34 (79.1)	0.087 (0.037–0.205)	<0.001

Abbreviation: PRLV, positional relation between lesions and V20 area.

### Diagnostic criteria

The diagnosis of IP was confirmed by microbiological examination which included blood culture and sputum culture (limited to high-quality specimens, defined as ≤10 epithelial cells and ≥25 white blood cells per low-power field). The diagnosis of ARP was confirmed by three factors: (1) the microbiological examination returned negative results; (2) a short-term improvement in symptoms was observed after treatment with glucocorticoids; (3) changes in CT imaging mainly occurred in the exposed area.

### Indicators

(1) PCT and white blood cell (WBC) count were serum and blood cell indicators, respectively. (2) Clinical characteristics included age, sex, pathological type, tumor stage, and physical status (PS) score. (3) Mean lung dose (MLD), lung volume receiving ≥20 Gy (V20), planning target volume (PTV) dose, and positional relation between lesions and V20 area (PRLV) (>50% inflammatory lesions in the range of V20 was referred as ‘inside V20’ and others as ‘outside V20’) were the radiophysical parameters.

### Statistical analysis

Univariate and multivariate analyses were performed using logistics regression. The dependent variables were binary ones, in which ARP was assigned a value of 1 and IP a value of 2. Among the independent variables, binary variables were assigned 1 and 2. The units of continuous variables PCT and C-reactive protein (CRP), WBC, MLD, and PTV dose, and V20 were μg/l, 10^9^/l, Gy, and 1%, respectively. The linear relationship between continuous variables and the logit values of dependent variables was verified using the Box–Tidwell test. The Spearman correlation analysis was used to calculate the correlation coefficients between continuous variables to avoid multicollinearity, and the collinearity test was performed in multivariate analysis to calculate the variance inflation factors. Based on the results of multivariate regression analysis, the nomogram was depicted to visualize the model. The discrimination, accuracy, and practicability of the model were evaluated using the receiver operating characteristic (ROC) curve, calibration curve, and decision curve analysis (DCA), respectively. Statistical analyses were performed with R software (version 3.5.3) and SPSS (version 22.0). All tests were two sided. A value of *P* less than 0.05 was considered significant.

## Results

### Univariate analysis of factors related to the diagnosis of ARP

Univariate analyses of categorical data, such as age, gender, pathological type, tumor stage, PS score, and PRLV, were performed. The results showed that only PRLV was significantly associated with ARP (OR = 0.087; 95% confidence interval (CI) = 0.037–0.205; *P*<0.001) (Table 1). The Box–Tidwell method was adopted to calculate the interaction between each continuous variable and its Ln value. The results revealed that the *P*-value of each indicator was greater than 0.05, suggesting a linear relationship between the continuous variables and the logit value of the dependent variable. The *P*-value of PCT, CRP, WBC, MLD, PTV dose, and V20 was 0.0567, 0.596, 0.097, 0.461, 0.442, and 0.358, respectively. The univariate analyses of continuous variables revealed that PCT, CRP, WBC, MLD, and V20 were associated with ARP (*P*<0.05), while PTV dose had a marginal correlation with RP (*P*<0.10) (Table 2).

**Table 2:** Univariate analysis of continuous variables

Variable	ARP median (IQR)	IP median (IQR)	*OR* (95%CI)	*P*-value
PCT	0.44 (0.30–0.90)	0.97 (0.65–1.51)	2.451 (1.413-4.252)	0.001
CRP	85.5 (47.3.5–124.5)	103.0 (75.0–137.3)	0.994 (0.988–0.999)	0.030
WBC	7.3 (5.9–8.5)	7.3 (6.6–10.2)	0.872 (0.776–0.981)	0.022
MLD	16.6 (13.7–19.4)	13.2 (11.6–16.2)	1.001 (1.000–1.002)	0.001
PTV dose	56.0 (52.0–60.0)	56.0 (50.0–60.0)	1.038 (0.997–1.081)	0.069
V20	28.0 (24.0–32.0)	26.0 (21.0–30.0)	1.054 (1.009–1.102)	0.018

### Correlation analysis of continuous variables

The Spearman correlation analysis showed a significant correlation between MLD and V20 (correlation coefficient: 0.763, *P*<0.001). The PTV dose correlated with MLD (correlation coefficient: 0.672, *P*<0.001) (Table 3).

**Table 3:** Correlation analysis of continuous variables

Variable	PTV dose	MLD	V20	PCT	CRP	WBC
PTVdose	1	0.672*	0.492*	0.08	0.111	0.067
MLD	0.672*	1	0.763*	−0.029	−0.138	0.029
V20	0.492*	0.763*	1	−0.056	−0.092	−0.036
PCT	0.08	−0.029	−0.056	1	0.357*	0.074
CRP	0.111	−0.138	−0.092	0.357*	1	0.041
WBC	0.067	0.029	−0.036	0.074	0.041	1

* *P*-value less than 0.01.

### Multivariate analysis of factors related to the diagnosis of ARP

**Figure 1:**
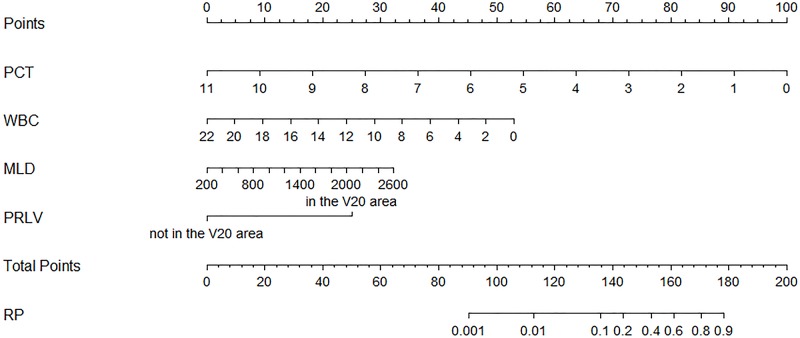
Nomogram of differential diagnosis of ARP

Variables with a *P*-value <0.10 in the univariate analysis were enrolled in the multivariate analysis. As the Spearman correlation analysis indicated a significant correlation between MLD and V20 (correlation coefficient: 0.752, *P*<0.001) and between MLD and PTV dose (correlation coefficient: 0.557, *P*<0.001), MLD with a minimum *P*-value was selected in the multivariate analysis. The results revealed that PRLV (*P*<0.001), PCT (*P*=0.014), WBC (*P*=0.009), and MLD (*P*=0.009) were independent diagnostic factors (Table 4). At the same time, the collinearity test demonstrated that the variance inflation factor of PRLV, PCT, WBC, and MLD was 1.097, 1.074, 1.103, and 1.062, respectively, all of which were smaller than 10 and suggested the absence of collinearity. Based on the multivariate logistic regression model, a visuable nomogram was developed, which is shown in Figure 1.

**Table 4:** Differential diagnosis related multivariate analysis

Variable	Regression coefficient	OR (95% CI)	*P*-value
PRLV (In V20 area vs Out of V20 area)	2.595	13.402 (4.837–37.130)	<0.001
PCT (μg/l)	−0.911	0.402 (0.195–0.830)	0.014
CRP (mg/l)	−0.001	0.999 (0.990–1.008)	0.816
WBC (10^9^/l)	−0.248	0.781 (0.649–0.939)	0.009
MLD (Gy)	0.001	1.001 (1.000–1.002)	0.009

**Figure 2:**
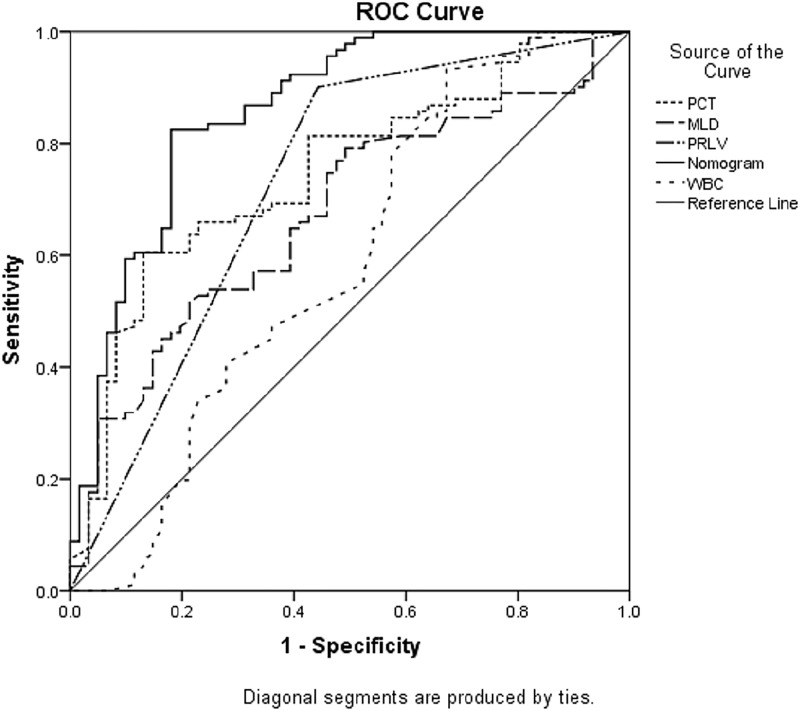
ROC curves for PRLV, PCT, WBC, and MLD

**Figure 3:**
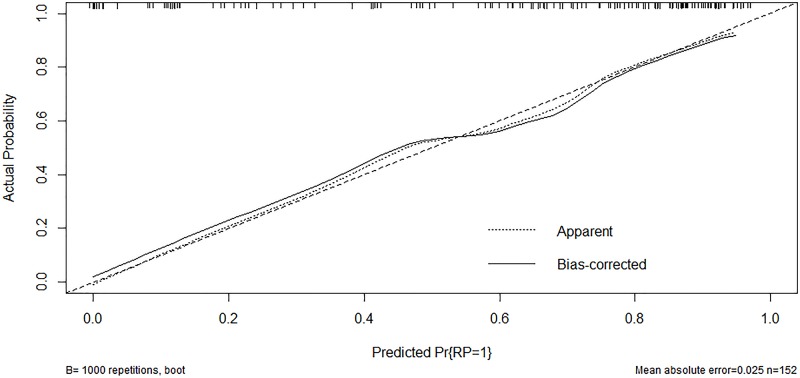
Calibration curve of the ARP prediction model

**Figure 4:**
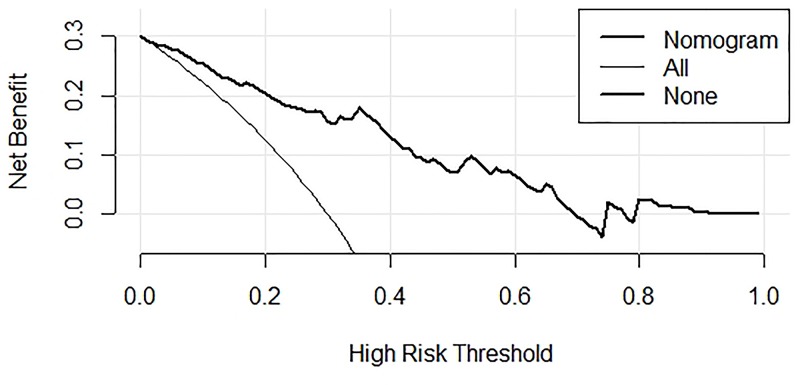
Decision curve of nomogram graph

The ROC curves of PRLV, PCT, WBC, MLD, and the regression model are shown in Figure 2. The area under the curve (AUC) of the model was 0.861 (95% CI: 0.795–0.911), which was significantly better than that of each single factor (PRLV: 0.729, 95% CI: 0.651–0.798; PCT: 0.747, 95% CI: 0.670–0.814; MLD: 0.674, 95% CI: 0.594–0.748; WBC: 0.586, 95% CI: 0.503–0.665). In addition, the model showed higher sensitivity and specificity (82.4 and 82.0%, respectively) (Table 5). The calibration curve showed that the diagnostic results using the present model were highly consistent with the actual diagnostic results (Figure 3). DCA showed a satisfactory positive net benefit of the model, which indicated the value of the model in the clinical differential diagnosis (Figure 4).

**Table 5:** Indicators of ROC curve for diagnostic evaluation of ARP

Variable	AUC (95%CI)	*P*-value	Cut-off point	Sensitivity	Specificity
PRLV	0.729 (0.651–0.798)	<0.001	In V20 area	90.1	55.7
PCT (μg/l)	0.747 (0.670–0.814)	<0.001	0.487	60.4	86.9
WBC (10^9^/l)	0.586 (0.503–0.665)	0.088	10.0	93.4	32.8
MLD (Gy)	0.674 (0.594–0.748)	<0.001	16.45	51.7	78.7
Nomogram	0.861 (0.795–0.911)	<0.001	0.508	82.4	82.0

## Discussion

The present study was novel in establishing a model for the differential diagnosis of ARP and IP. A total of 152 patients with lung cancer who developed pneumonitis (91 with ARP and 61 with IP) within 6 months after radiotherapy were included. The univariate analysis showed that PRLV, PCT, CRP, WBC, MLD, and V20 were associated with ARP, while PTV dose marginally correlated with ARP. After excluding collinearity indicators, multiple indicators were included in the multivariate analysis. The results revealed that PRLV, PCT, and MLD were independent diagnostic factors, and WBC marginally correlated with ARP. Subsequently, the nomogram was obtained using the regression model based on the multivariate analysis. The ROC curve showed that the AUC of the model was 0.861 (95% CI: 0.795–0.911), which was significantly better than that of every single factor. The model had high sensitivity and specificity (82.4 and 82.0%, respectively). The calibration curve and the DCA curve revealed that the model had high diagnostic accuracy and positive net benefit, which indicated the value of the model in the clinical differential diagnosis.

Early diagnosis of ARP was critical for treating ARP, as absolutely different strategies may arise based on different diagnostic results, while wrong treatment can lead to serious consequences [7]. However, early accurate diagnosis is a tough task. The diagnosis of ARP is an exclusive method in which the difficulty is to differentiate it from IP. The pathogen-based test is more likely to accurately diagnose IP, which can further help to determine ARP. However, it takes a long time to obtain a pathologic diagnosis, and it is difficult to obtain relevant high-quality specimens, such as bronchial lavage fluid, indicating that pathogenic examination is not suitable for early diagnosis. To address this problem, serological PCT and hematological indicator WBC were introduced in the present study.

PCT is an effective biomarker for the diagnosis and detection of bacterial infections, and is currently widely used in clinical practice [8–10]. PCT can also help clinicians to determine non-infectious diseases. A study to determine the cause of fever in patients with advanced urinary tract tumors showed that PCT could be used to differentiate infectious fever and tumor-derived heat [11]. Another study compared the PCT level between patients suffering from infectious fever and non-infectious fever after chemotherapy for lung cancer. It found that the PCT in patients with non-infectious fever was significantly lower than that in patients with infectious fever [12]. As the nature of ARP was also non-infectious, it was inferred that PCT might play a role in the differential diagnosis of ARP and IP. In the present study, both univariate and multivariate analyses confirmed that PCT was an independent predictor of ARP. Ultimately, PCT was also integrated into the differential diagnosis model. In another study, attempts were made to construct a scoring system for the diagnosis of ARP, which also included PCT; the results were consistent with those of the present study [13]. CRP is a traditional inflammatory indicator, but it is not specific in distinguishing between infectious and non-infectious diseases [14]. In the present study, it was not shown to be able to differentiate ARP from IP.

In the present study, WBC was another factor included in the model. The multivariate analysis showed that WBC correlated with ARP, and the probability of diagnosing APR seemed to increase with the reduction in WBC, indicating the existence of a relatively high level of WBC in most patients with IP and a lower level of WBC in the patients with ARP. These results were consistent with those reported by Ramella et al. [13], in which increased granulocyte count was also included as a factor. Another study found that the WBC count was associated with radiotherapy-induced changes in imaging [15]. However, patients with granulocyte deficiency accompanied by fever were not included in the present study. These patients were highly likely to be infected. Hence, it was necessary to be alert to cases with granulocyte deficiency accompanied by a fever in clinical practice so as to avoid misdiagnosis [16].

In the present study, another important factor included in the diagnosis model was the radiophysical parameter MLD. MLD itself was not a diagnostic indicator, but a predictive indicator of ARP. In a series of studies exploring the risk factors associated with ARP, multiple radiophysical parameters were confirmed to be associated with the occurrence of ARP, including MLD, V20, and so on [17]. The inclusion of these predictors can contribute to locking in high-risk patients with ARP; and together with other factors, it can improve the accuracy of differential diagnosis, like the role of cirrhosis, hepatitis, and other risk factors in diagnostic criteria for primary liver cancer. The results of the present study also confirmed that MLD, as an independent predictor, was one of the multiple diagnostic factors, which differed from previous studies that did not report the diagnostic value of radiophysical parameters [13]. This might be because previous studies enrolled only a small number of samples and did not include radiophysical parameters along with the clinical parameters in their multivariate analysis.

The present study had many limitations. First of all, this was a retrospective study, and hence relevant bias was inevitable. Relevant prospective studies need to be conducted in the future to validate and improve the present differential diagnosis model. Second, the sample size was limited, and therefore the established model was only verified internally rather than externally. External verification needs to be performed in future prospective studies. In addition, the present study focused on the differential diagnosis of ARP and IP, in which the majority of patients with IP were infected with bacteria and a small number of patients were infected with fungi. Hence, fungal infection-related markers were not included in the analysis due to insufficient information. It is speculated that the inclusion of these factors may further improve the accuracy of the model because laboratory indicators related to fungal infections, such as G-test, have definite clinical value, which needs to be confirmed in future studies [18].

In summary, the logistics regression multivariate analysis revealed that PRLV, PCT, and MLD were independent diagnostic factors, and WBC marginally correlated with ARP. Based on these results, the nomogram was successfully depicted, and the internal verification prompted the validity of the model. These results need verification and improvement using prospective studies.

## Abbreviations

ARP: acute radiation pneumonitis
AUC: area under the curve
CI: confidence inerval
CRP: C-reactive protein
CT: computed tomography
DCA: decision curve analysis
IP: infectious pneumonia
IQR: interquartile range
MLD: mean lung dose
OR: odds ratio
PCT: procalcitonin
PRLV: positional relation between lesions and V20 area
PS: physical status
PTV: planning target volume
ROC: receiver operating characteristic
V20: lung volume receiving ≥20 Gy
WBC: white blood cell
